# Relationship between the Central and Regional Pulse Wave Velocity in the Assessment of Arterial Stiffness Depending on Gender in the Geriatric Population

**DOI:** 10.3390/s23135823

**Published:** 2023-06-22

**Authors:** Iwona Jannasz, Tadeusz Sondej, Tomasz Targowski, Małgorzata Mańczak, Karolina Obiała, Andrzej Piotr Dobrowolski, Robert Olszewski

**Affiliations:** 1 Department of Geriatrics, National Institute of Geriatrics Rheumatology and Rehabilitation, 02-637 Warsaw, Poland; iwona.jannasz@spartanska.pl (I.J.); tomasz.targowski@spartanska.pl (T.T.); 2 Faculty of Electronics, Military University of Technology, 00-908 Warsaw, Poland; andrzej.dobrowolski@wat.edu.pl; 3 Gerontology, Public Health and Education Department, National Institute of Geriatrics Rheumatology and Rehabilitation, 02-637 Warsaw, Poland; m.manczak@op.pl (M.M.); obialsky@gmail.com (K.O.); robert.olszewski@spartanska.pl (R.O.); 4 Department of Ultrasound, Institute of Fundamental Technological Research Polish Academy of Sciences, 02-106 Warsaw, Poland

**Keywords:** pulse wave velocity, arterial stiffness, cardiovascular risk, geriatrics, gender differences, photoplethysmography, multi-site PWV

## Abstract

Artery stiffness is a risk factor for cardiovascular disease (CVD). The measurement of pulse wave velocity (PWV) between the carotid artery and the femoral artery (cfPWV) is considered the gold standard in the assessment of arterial stiffness. A relationship between cfPWV and regional PWV has not been established. The aim of this study was to evaluate the influence of gender on arterial stiffness measured centrally and regionally in the geriatric population. The central PWV was assessed by a SphygmoCor XCEL, and the regional PWV was assessed by a new device through the photoplethysmographic measurement of multi-site arterial pulse wave velocity (MPPT). The study group included 118 patients (35 males and 83 females; mean age 77.2 ± 8.1 years). Men were characterized by statistically significantly higher values of cfPWV than women (cfPWV 10.52 m/s vs. 9.36 m/s; *p* = 0.001). In the measurement of regional PWV values using MPPT, no such relationship was found. Gender groups did not statistically differ in the distribution of atherosclerosis risk factors. cfPWV appears to be more accurate than regional PWV in assessing arterial stiffness in the geriatric population.

## 1. Introduction

In recent decades, we have seen an increase in life expectancy, but cardiovascular diseases (CVD) are still the leading cause of morbidity and mortality [1,2]. Cardiovascular risk (CVR) is a result of many interacting risk factors. Commonly recognized classical risk factors for CVD include age, previous family history of heart disease, and modifiable risk factors, such as hypertension, hyperlipidemia, smoking, diabetes, and obesity [3]. In most cases, these factors lead to the formation of atherosclerosis—the main cause of CVD. Atherosclerosis is a progressive process characterized by the collection of lipids, inflammatory cells, and fibrous elements in the walls of arteries, resulting in progressive narrowing and stiffening of the arteries [4]. Artery stiffness increases with age, which is why vascular aging is a risk factor for CVD [5]. An increase in arterial stiffness is a major cause of an increase in systolic blood pressure (SBP) and pulse, as well as a decrease in diastolic blood pressure (DBP) during the aging process [6].

### 1.1. Pulse Wave Velocity

The measurement of the pulse wave velocity (PWV) between the carotid artery and the femoral artery, which is defined as the carotid–femoral pulse wave velocity (cfPWV), is considered the gold standard for arterial stiffness assessment [7,8]. Over the years, cfPWV measurement has been used for the assessment of the risk of cardiovascular events in the population of healthy people. Patients with a specific disease entity were also assessed, and PWV was compared with other recognized CVR factors [9]. cfPWV has a predictive value for CVD that goes beyond traditional CV risk factors in the general population among patients with various diseases. It may also be useful to stratify the risk of atherosclerosis. Various studies have reported that PWV is a powerful predictor of CV events as well as all-cause mortality that may occur in the future [10].

In addition to the gold standard, which is cfPWV, other parameters resulting from pulse wave analysis (PWA) are often the augmentation index (Aix) or augmentation pressure (AG). AG, defined by the height of the late systolic peak (P1) above the inflection (P2), is the contribution that wave reflection makes to systolic arterial pressure. Aix is calculated as AG divided by pulse pressure (PP) x100 [7].

### 1.2. Regional Pulse Wave Velocity

As the function and diameter of arterial vessels decreases, the composition of the arterial wall changes from the central aorta towards the periphery; centrally, the center of a large elastic artery has an ultrastructure of concentric elastic lamellae, intersected by layers of connective tissue, which contain smooth muscle cells. This microstructure gradually disappears, and the increased content of smooth muscle cells in medium-sized and especially smaller vessels takes over [11]. Therefore, apart from the central pulse wave velocity, the importance of the newly examined element increases in the places of regional measurement, including, apart from the elastic aorta, also the greater part of the muscular arteries [12].

### 1.3. Cardiovascular Risk Factors

Epidemiological studies show that the average life expectancy of men is lower than that of women [13]. In aging adults, gender is considered a significant risk factor for occurrence and curing of CVD [14]. A study on Europeans aged 50 years and over emphasized that the main mortality risk factors were: older age, poor self-rated health, activities of daily living (ADL) deficits, male gender, lower cognition, comorbidity, and presence of depressive symptoms [15]. However, it has not been proven what exactly affects the frequency and the presence of classic risk factors for CVD, which is associated with increased mortality in older men. There is little research explaining why gender, regardless of other classic cardiovascular risk factors, can be a determining factor in life expectancy [16].

## 2. Materials and Methods

### 2.1. Design and Participants

The data were collected during the Geriatric Arterial Stiffness Measurement Evaluation study (GAME). This prospective cohort part of the study aims to investigate the influence of gender differences on markers of arterial stiffness. The criterion for inclusion of patients was age over 60 years. The study group included 118 consecutives patients (mean age 77.2 ± 8.1 years) hospitalized in the Department of Geriatrics of the National Institute of Geriatrics, Rheumatology, and Rehabilitation from December 2018 to July 2019.

The study was designed to be observational and not interventional; we decided the PWV result could not influence a change of therapy. The majority of patients in the study were elderly patients with multiple diseases who received standard, continuous pharmacotherapy in accordance with the latest guidelines and the best medical knowledge, also regarding hypertension or atherosclerosis, if necessary. Given that the patients were in a stable condition (diagnostic hospitalizations), their treatment was established, and blood pressure values were adjusted before hospitalization—which also implies before PWV measurements.

### 2.2. Exclusion Criteria

Exclusion criteria were active cancer, lack of limbs, and advanced dementia process preventing collaboration with the investigator’s recommendations.

### 2.3. Consent of the Bioethics Committee

This study’s protocol complies with the ethical guidelines of the 1975 Declaration of Helsinki and has been approved by the Bioethics Committee at the National Institute of Geriatrics, Rheumatology, and Rehabilitation in Warsaw. Before inclusion in the study, all participants were made to provide written informed consent.

### 2.4. Measurement of cfPWV

**Figure 1:**
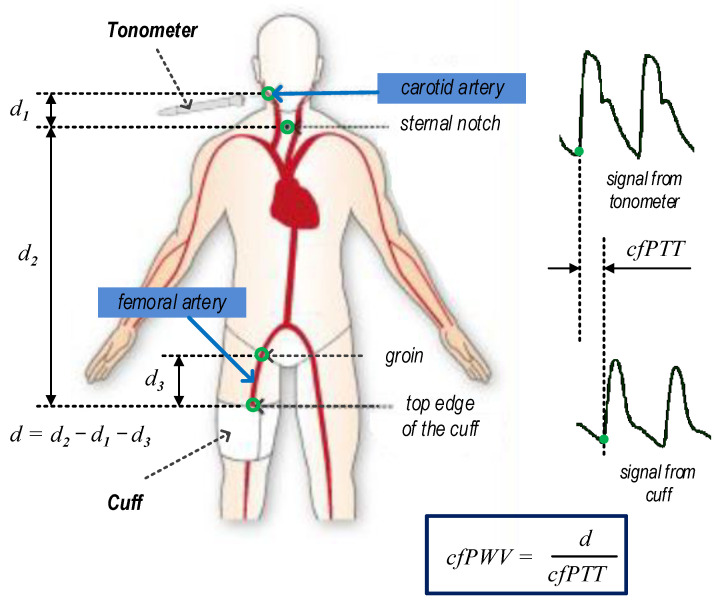
Principle of cfPWV measurement with the SphygmoCor XCEL device.

The cfPWV value was assessed by a SphygmoCor XCEL from ATCOR [17]. The SphygmoCor XCEL device has been validated as per the ARTERY PWV validation guidelines [18]. Although other devices are known, the SphygmoCor is the most widely used and considered the gold standard technique [19]. The principle of cfPWV measurement with the SphygmoCor XCEL device is shown in Figure 1.

**Figure 2:**
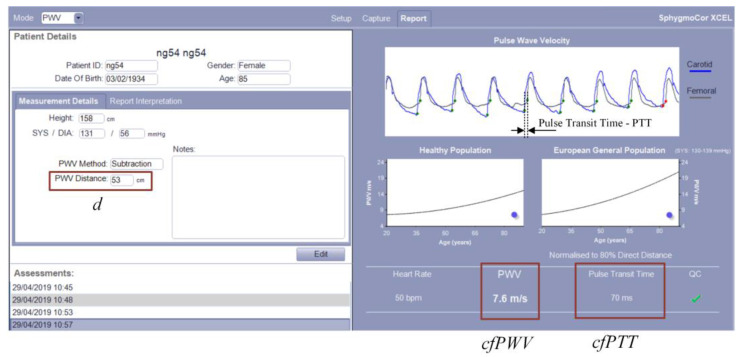
An example screenshot of cfPWV measurement with the SphygmoCor XCEL.

Measuring cfPWV with the SphygmoCor XCEL apparatus simultaneously detects a carotid pulse by applanation tonometry and a femoral pulse by volumetric displacement with a cuff around the upper thigh [20]. Then, the device measures the pulse transit time (cfPTT) between the diastolic feet of the carotid and femoral pulse. The path length (distance—d) was calculated by subtracting the distance between the carotid artery measurement site and sternal notch (carotid–notch) from the distance between the femoral artery site and the sternal notch (femoral–notch), all measured directly with a tape measure with a reading accuracy of ±0.5 cm. cfPWV was calculated as follows: cfPWV (m/s) = distance/cfPTT. An example screenshot of cfPWV measurement with the SphygmoCor XCEL and additional explanations is shown in Figure 2.

After entering the data about the participant (patient), taking the distance measurements (according to Figure 1), and taking the measurement, the registered signal waveforms from the carotid and femoral arteries, the resulting cfPTT and cfPWV values are displayed on the right side of the window. In addition, the measurement quality index (QC), the heart rate, and the obtained PWV result against the background of the healthy and European general population are displayed.

During the measurements, it was very useful to view the signals from the carotid and femoral arteries. Thanks to this, it was possible to reject noisy or poor-quality measurements.

**Figure 3:**
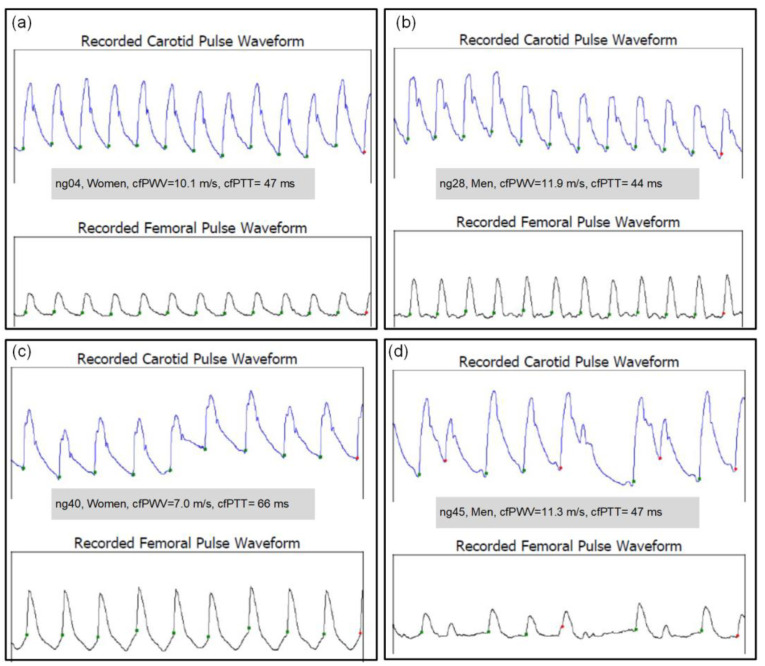
Representative carotid and femoral pulse waveforms for calculating the cfPWV recorded with the SphygmoCor XCEL (for 4 participants with ID = (**a**) ng04, (**b**) ng28, (**c**) ng40 and (**d**) ng45).

It is worth noting that the measured signals are usually different for each participant. Representative signals (carotid and femoral pulse waveform) for 4 participants are shown in Figure 3. 

Signal graphs come from reports generated by the SphygmoCor XCEL software (version 1.3.2.18). The shown examples of pulse waveforms have different amplitudes, shapes, and durations. These parameters are related to the individual characteristics of the participants. However, this does not have a significant impact on the result because the cfPWV is calculated on the basis of the pulse wave propagation time according to the validated algorithms of the SphygmoCor XCEL apparatus.

### 2.5. Measurement of Multi-Site PWV

**Figure 4:**
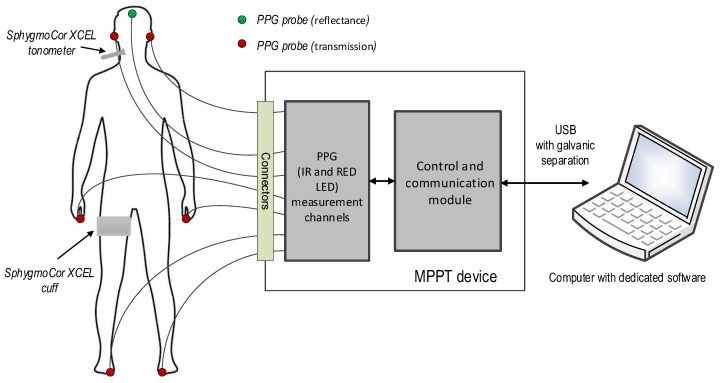
Block diagram of the MPPT system and location of PPG sensors for regional PWV measurement.

For multi-site arterial pulse wave velocity measurements, we used a custom-made system called MPPT. This system measures the regional PWV. To measure PWV, it uses PPG (photoplethysmographic) sensors located at different sites. This system was described in detail in [21]. For multi-site PWV measurement, we used 7 PPG sensors as shown in the MPPT configuration diagram (Figure 4).

In addition, localization of the SphygmoCor XCEL sensors (tonometer and cuff on right body site) is shown in the block diagram. Multi-site regional PWV measurement with the MPPT device was described in detail in our previous work [22]. The MPPT device synchronously measures several PPG signals from different locations (forehead, ears, fingers, and toes) and then calculates the PWV based on the pulse transit time and distance between the sternal notch and PPG sensors. A reflective sensor was used on the forehead and a transmission sensors on other locations. For PWV calculations we used signals from an IR diode (wavelength 905 nm). The MPPT device was connected to a computer via a USB interface with galvanic separation. Dedicated computer software was responsible for control, online data transfer, and visualization of signals as well as data archiving. Signal processing and calculation of PWV were performed offline in the MATLAB environment (version R2019a). All distances for regional PWV assessment were obtained directly with a tape measure with a reading accuracy of ±0.5 cm.

The MPPT system calculated the regional PWV from the PPG signals measured at a sampling frequency of 1 kHz. The beginning of the pulse wave for each of the pulses was determined by the intersecting tangent method, according to [22].

**Figure 5:**
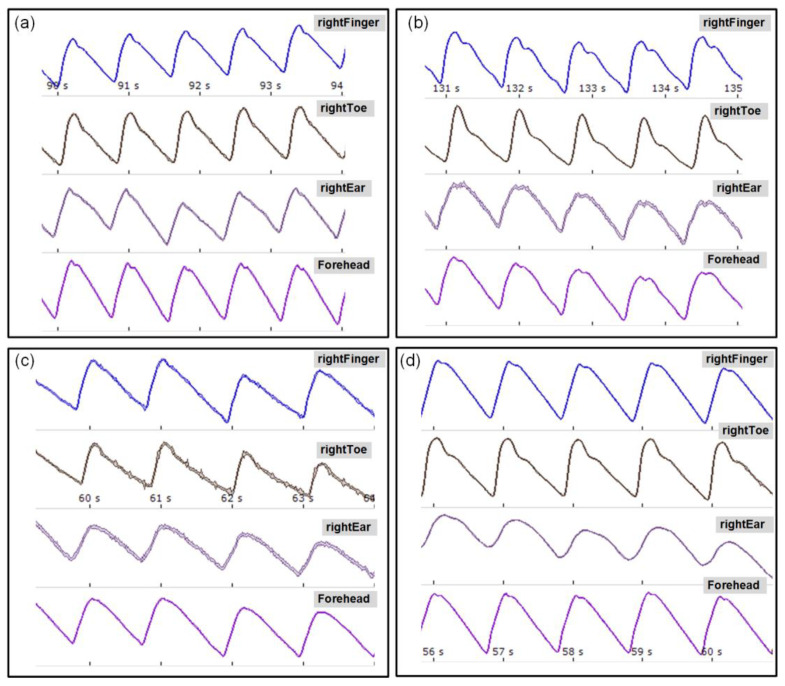
Representative signal plots for calculating the regional PWV recorded with the MPPT apparatus (for 4 participants with ID: (**a**) ng04, (**b**) ng28, (**c**) ng40 and (**d**) ng45).

Representative PPG signals (synchronously acquired from the right finger, toe, ear, and forehead) measured by MPPT devices for 4 participants are shown in Figure 5.

The presented examples of PPG pulse waveforms differ from each other, especially in shape, depending on the site of measurement and the individual characteristics of the participant. However, this does not have a significant impact on the result because, as for the SphygmoCor XCEL, the regional PWV is calculated on the basis of the pulse wave propagation time according to the validated algorithms of the MPPT apparatus.

### 2.6. Measurement Protocol

For more accurate results, in our study, we took the measurements according to the same procedure for each participant. After a minimum 15 min rest and after informing the participant about the purpose of the study and obtaining their signed consent, the blood pressure in the left brachial was measured using the SphygmoCor XCEL in pulse wave analysis (PWA) mode. This measurement was performed in the standard sitting position, and its purpose was to determine the brachial (bSBP, bDBP) and the aortic (aSBP, aDBP) blood pressure. Next, the participant assumed a supine position on a medical settee and rested for about 15 min. During this time, the SphygmoCor XCEL and MPPT apparatus sensors were connected, distances were measured, and signals were checked. Subsequently, the main measurement was performed, lasting exactly 15 min. It should be stressed that the measurements with the SphygmoCor XCEL were performed simultaneously with the measurements with the MPPT apparatus. The MPPT measured the PPG signals continuously for 15 min. At the same time, a minimum of three cfPWV measurements were made at an interval of approximately 3 min. All measurements were performed by the same operator, during working days, from Monday to Friday, from about 10 a.m. to 1 p.m., in a separate and quiet room, with an ambient temperature of about 22–24 °C.

The final regional PWV was calculated offline for each participant as the mean of the 15 min recording. Likewise, for each participant the average of the all cfPWV readings was calculated.

### 2.7. Analysis

Statistical analysis was performed using Matlab, R environment, and Statistica v.13. *p* < 0.05 was considered statistically significant. For continuous variables, the normal distribution was checked using the Shapiro–Wilk test. Student’s *t*-test was used to compare normally distributed continuous variables, and data were reported as means with standard deviations. The Mann–Whitney U test was used to compare non-normally distributed variables, and data were reported as medians and interquartile ranges. The Pearson’s chi-square test or chi-square test with Yates correction was used to compare discrete variables depending on the expected values. Linear correlation analysis between PWV and continuous variables was performed, and Pearson’s r coefficient was determined. Variables with Pearson’s correlation coefficients higher than 0.3 (*p* < 0.05) were included in the multivariable regression model. Brachial systolic blood pressure (bSBP) was chosen as a representative of the strongly correlated variables relating to blood pressure.

## 3. Results

### 3.1. Characteristics of the Study Group

Table 1 shows the clinical characteristics of the 118 subjects (35 males and 83 females) in the GAME study. Both gender groups were quite homogeneous in terms of the distribution of comorbidities like hypertension, diabetes mellitus, metabolic syndrome, heart failure, and chronic obstructive pulmonary disease.

Patients of both groups did not statistically differ in the values of age; blood tests such as LDL-C, TG, FPG, eGFR, and TSH; blood pressure values (SBP, DBP, MAP—measured on the brachial artery as well as the central one—and aortic pressure); or anthropometric measurements such as upper-arm circumference and lower-leg circumference. BMI and TC were higher in women, reaching a statistically significant *p*-value (*p* = 0.05). In comparison with females, males exhibited a significantly lower HDL-C level (*p* < 0.001), higher uric acid level (*p* < 0.001), and higher NTproBNP concentration (*p* = 0.047). Women had higher inflammation parameters (CRP *p* = 0.047; ESR *p* = 0.050). Men were characterized by statistically significantly higher values of cfPWV than women (cfPWV median 10.52 m/s vs. 9.36 m/s, respectively; *p* = 0.001).

### 3.2. Gender Differences in the Analysis of the Impact of cfPWV on Selected Atherosclerosis Risk Factors and Comorbidities

Table 2 shows the correlation coefficients between cfPWV and selected parameters.

The highest correlations in the entire group were found for systolic arterial pressure, both peripheral bSBP (r = 0.443) on the brachial artery as well as systolic pressure of the central estimated aortic measurement aSBP (r = 0.411). Moreover, all pressure parameters (bDBP, bMAP, aDBP, aPP, and aMAP) showed a significant relationship with PWV. In the whole group, other significant parameters associated with arterial stiffness were patients’ age (r = 0.341; *p* < 0.001), degree of heart failure expressed as elevated concentration of NTproBNP (r = 0.347; *p* < 0.001), and uric acid level (r = 0.339; *p* < 0.001). cfPWV growth was sometimes observed to have a different potency between the groups. Sometimes the differences were discreet, as in aMAP, which increases cfPWV in both women and men; the correlation coefficient is higher in women, but the difference does not reach statistical significance. Our study also presents an analysis of the relationship with age—which significantly correlates in women (r = 0.429; *p* < 0.001)—and its importance has not been registered in the group of men (r = 0.193; *p* = 0.265).

Table 3 shows the results of multivariable analysis examining the influence of various parameters on cfPWV in the whole group and the gendered subgroups.

The most significant parameters in the whole group were two modifiable factors: systolic blood pressure (β 0.398; *p* < 0.001) and uric acid value (β 0.172; *p* = 0.034), and two non-modifiable ones: male gender (β 0.251; *p* = 0.003) and age (β 0.250; *p* = 0.003). In the multivariable regression analysis in the group of women, apart from the values of systolic blood pressure (β 0.355; *p* < 0.001), age (β 0.276; *p* = 0.006), and uric acid level (β 0.240; *p* = 0.010), the value of NTproBNP (β 0.208; *p* = 0.034) also had a significant impact on cfPWV. Meanwhile, in the multivariable analysis concerning the group of men, only the value of systolic blood pressure (β 0.394; *p* = 0.010) was significant.

### 3.3. Multivariable Regression—Comorbidities and Gender

The multivariate regression analysis of the influence of comorbidities, mainly cardiovascular, included in Table 4 was supplemented with the male gender, a recognized cardiovascular risk factor which is not correlated with any of the analyzed diseases. In our analysis, male gender significantly (β 0.251; *p* = 0.005) influences the increase in PWV; only diabetes (β 0.279; *p* = 0.002) is a stronger factor and is characterized by a greater influence than the presence of hypertension (β 0.196; *p* = 0.029).

### 3.4. Analysis of Multi-Site Regional PWV by Gender

Table 5 presents the results of measurements of regional PWV taken at the six body sites discussed above, broken down by gender. In contrast to the central cfPWV measurement, no statistically significant differences between the sexes were noted in any of the regional measurements.

### 3.5. Comparison of Central and Regional PWV

Table 6 compares the central PWV with the regional PWV. The difference in mean PWV (mean difference) values was determined. In the overall analysis, the differences between cfPWV and regional PWV are noteworthy, with generally higher values for mean central PWV. In the group of men, each of the regional measurements is statistically significantly lower than the cfPWV value.

## 4. Discussion

### 4.1. Results

To the best of our knowledge, this is the first study that demonstrates differences in cfPWV between genders in nearly all homogeneous patients in terms of classic comorbidities such as hypertension, diabetes mellitus, metabolic syndrome, chronic obstructive pulmonary diseases, and heart failure among Polish geriatric patients and shows differences in the impact of individual risk factors on the cfPWV value in gender groups. There are some studies that have assessed the relationship between arterial stiffness and gender, but most of them have been conducted in the younger population [23,24,25,26,27]. No significant difference was found for PWV, arterial age, and augmentation index in an analysis of gender and arterial stiffness among smokers (mean age 38). In addition, differences between smoking pack-year values (18.5 pack-years in male and 7.5 pack-years in female) between sexes that increase arterial stiffness were emphasized [23]. In participants with prehypertension (mean age 59.76 + 12.37) selected from the BEST study, males had higher PWV than females (10.89 vs. 10.33 m/s, respectively). However, differences in the distribution of other CV risk factors were observed, such as: 1) age, BMI, FPG, UA, and homocysteine being higher in males compared with females, and 2) TC, HDL-C, and LDL-C being higher in females [24]. In a study conducted among the Tallinn population aged 20–65, a higher PWV was observed in hypertensive men aged equal to or above 50 years, as well as in hypertensive women with diabetes and in apparently healthy women with increased apolipoprotein B [25]. Another research work of carotid stiffness measured with ultrasound echo-tracking presented no significant difference in PWV-β between genders in the age group 54.7 ± 10.6 years. Gender might play a modulatory role in the interconnection between arterial stiffness and some risk factors, where there appears to be a stronger relationship between stiffness and heart rate in men and pulse pressure in women [26]. In a study with morbidly obese patients (BMI of ≥40 kg/m^2^ or a BMI of ≥35 kg/m^2^ and obesity-related comorbidity), aged 18 to 65 years, the median PWV was significantly higher in men than women (7.3 m/s (IQR 6.6–8.0) and 6.8 m/s (IQR 5.9–8.0), respectively); the lower PWV in women appears to diminish in morbidly obese women after menopause [27]. The study emphasized the role of different hormonal balances, including the protective effect of estrogens in premenopausal women compared to young men. However, the initially protective role of female sex hormones in combination with the subsequent acceleration of increased cardiovascular risk remains unclear [28,29]. The advantage of our study is the age of the surveyed population, as they were geriatric patients. Worthy of note is that this group is seldom included in other studies. The age of the group in our study reduces the influence of sex hormones because the women we examined were postmenopausal. This allows to objectively compare their cardiovascular risk to men of the same age. There are single reports about the negative influence of male gender on the advancement of the atherosclerotic process. Male gender was an independent predictor of re-peripheral vascular interventions in a study assessing long-term clinical outcomes in patients with chronic total occlusions of infrainguinal lower-limb arteries [30]. As far as we know, only a few studies concentrated on arterial stiffness in the elderly population. A Parisian geriatric study (mean age 87.1 ± 6.6) indicated that age and loss of autonomy were the best predictors of mortality, and aortic PWV was the major independent risk predictor for cardiovascular mortality, whereas systolic blood pressure or pulse pressure was not. Unfortunately, a gender analysis was not provided by the researchers [31]. One of the few studies among the elderly (over 80 years of age) assessing the subclinical markers of atherosclerosis, such as endothelial dysfunction and carotid thickness, presents the relationships between them and osteoporosis expressed in decreased bone mass. However, this study did not assess the difference between the sexes [32]. The main result of our GAME study is that PWV is higher in men than in women, despite the similar distribution of other classic CV risk factors (age, blood pressure, LDL cholesterol, and kidney function). Our next task is to look for other discrete factors that can affect increases in PWV in men, which can lead to earlier mortality and other complications of high arterial stiffness. We also analyzed the strength of the impact of individual parameters on increases in PWV in the entire patient group. The strongest factor affecting increases in arterial stiffness turned out to be systolic arterial pressure, both the peripheral value measured on the brachial artery and the central pressure values estimated by the SphygmoCor; following that were the average blood pressure values and age, as well as the NTproBNP values. Interestingly, in the whole group, the parameters of the lipid profile seem to be irrelevant for PWV increases, with only a slightly outlined negative correlation for HDL-C values. Meanwhile, analyzing the impact of specific factors in groups of men and women, we also find different relationships. In men, PWV (apart from SBP values that are significant for both sexes) is significantly affected by the values of triglycerides and glucose. In women (except for the discussed SBP), age, NT-proBNP, uric acid, and renal function (eGFR) have the most significant impacts. The above analysis allows us to suppose that groups of women and men should be analyzed separately, and one should look for different risk factors for arterial stiffness except for classical CVR.

The development of new methods of measuring arterial stiffness allows us to better understand the different components of stiffness as well as to estimate their impact on the real condition of arteries (possible to be fully unambiguous only in autopsy post mortem). [33] Research on arterial stiffness is still an ongoing issue. Attempts have been made to study the construct validity of a measure of PWV estimated from age and blood pressure (ePWV) [34].

Recently, the usefulness of photoplethysmography signals in medicine is being investigated [35]. Methods to take measurements continuously and across multiple body sites using photoplethysmography are also being developed, such as the comparison of overall agreement and repeated measures such as heart–finger PWV (hfPWV) and heart–toe PWV (htPWV). In [36], htPWV measurements were compared to oscillometric carotid–wrist PWV (cwPWV) and carotid–ankle PWV (caPWV) referent measurements in a group of 30 young people (24.6 ± 4.8 years). In a Czech study by L. Soukup [37] of 220 (age 21–71) normal, healthy, normotensive people who had no history of disease that had a major impact on PWV values and were not taking any related medications, the reliability of whole-body multi-channel bioimpedance to assesses pulse wave velocity and provide a reference value for measuring whole-body PWV was examined. In addition, a significant age-dependent PWV of the aorta was found in these values measured using the left carotid as the proximal artery. PWV values in the upper and lower limbs do not show a significant dependence on age. Disagreement of a single peripheral measurement of heart–finger pulse wave velocity in comparison to brachial–ankle pulse wave velocity were also noted in a Korean study of healthy adults (92 males and 93 females) of ages ranging from 20 to 66 [38]. Referring to the slightly different results in our study, it should be emphasized that we studied elderly people with multimorbidity, which increases the stiffness of the arteries, mainly the aorta. Most studies to date have been based on young or middle-aged healthy individuals. In our geriatric study, central PWV values, especially in men, were higher than regional values in every measurement. More research is needed, optimizing on a larger group of people and assessing long-term effects, to explain this relationship. Based on current knowledge, we can assume that a greater component of arterial stiffness is the aorta rather than other arteries of smaller caliber as well as intramuscular arteries. Regional measurements show that small arteries are likely to age similarly in men and women.

The advantage and novelty of our study is the group of geriatric patients in whom the atherosclerotic process is already developed, which allows for an objective assessment of arterial stiffness measurement methods.

### 4.2. Strengths and Limitations

However, certain limitations should also be acknowledged. First, most patients are women (83 women vs. 35 men), and therefore we were unable to obtain statistical significance in some relationships. The gender gap is caused by the predominance of women in the older population as well as the predominance of women among those hospitalized.

As we aimed at a population study among hospitalized people, every patient hospitalized in the Department of Geriatrics of the National Institute of Geriatrics, Rheumatology, and Rehabilitation from December 2018 to July 2019 was included in the study. The only exclusion criteria were active cancer, lack of limbs, and advanced dementia process preventing collaboration on the investigator’s recommendations.

The life expectancy of women in Poland is 8 years longer than that of men. In the analyzed period in the Department of Geriatrics of the National Institute of Geriatrics, Rheumatology, and Rehabilitation, which is comparable to the data of the Polish National Health Fund, women constitute about 65–70% of patients hospitalized in geriatric wards. We did not decide to study only a proportion of women to match their numbers with the men, as then the criteria for inclusion or non-inclusion of a specific man could be unclear. We hope that further, larger observational studies may be interesting, being typical studies of entire populations, e.g., cities or countries, and not taking into account the criterion of the need to hospitalize the patient.

The second is the lack of a cut-off point for elevated PWV. It is not described in the current literature, and our study group is too small to extrapolate values recognized by other scientific authorities (e.g., the 12 m/s value recognized as a risk factor for people with hypertension by the European Society of Cardiology [9]). We think that an interesting development of the current work will be prospective observations with an analysis of mortality and cardiovascular incidents in our group that we conducted.

Another limitation of this work is the heterogeneity among geriatric patients in this study. During the measurement process, we did not consider the individual differences of the subjects because our goal was to reproduce the PWV assessment among patients hospitalized in the Department of Geriatrics as authentically as possible. For this reason, we did not use inclusions for chronic diseases (such as diabetes, hypertension, or chronic kidney disease) because they constitute the overall clinical picture of the geriatric patient.

## 5. Conclusions

In conclusion, the result of the current GAME study shows that cfPWV is higher in men than women in the geriatric population. However, the reason for this relationship is still unclear and cannot be explained by the distribution of classical CV risk factors (age, systolic blood pressure, and total cholesterol) between genders. Because of the attempts made to reduce this important CVD risk factor in the elderly patient population, further studies aimed at deciphering the secret of increased arterial stiffness in men could be remarkably interesting. In addition, it is necessary to look at gender differences separately. In men and women, various factors affect increases in arterial stiffness and thus increase cardiovascular risk. Therefore, it is worth conducting gender-shield analyses as an introduction to personalized medicine. In addition, taking into account the less clearly differentiated regional PWV values obtained by MPPT, it should be assumed that the main factor affecting the stiffness of these arteries is the competitor of the elastic great central arteries. The main problem of arterial stiffness, and thus of all clinical consequences with the aging of the population, is atherosclerosis and calcification, mainly affecting the aorta and, to a much lesser extent, peripheral muscular arteries.

## Figures and Tables

**Table 1:** Comparative characteristics of gender groups.

	Women (*n* = 83)	Men (*n* = 35)	*p*-Value
cfPWV (m/s)	9.36 (8.28–10.63)	10.52 (9.18–11.65)	0.001
Age (years)	77 (72–83)	76 (69–86)	0.810
TC (mg/dL)	199 (165–226)	167 (135–224)	0.051
HDL-C (mg/dL)	63 (54–72)	51 (39–61)	<0.001
LDL-C (mg/dL)	107.8 (84.0–138.2)	98.4 (64.2–145.0)	0.208
TG (mg/dL)	111 (82–150)	107 (87–170)	0.874
FPG (mg/dL)	95 (87–109)	95 (88–107)	0.751
NTproBNP (pg/mL)	225.4 (128.6–410.0)	322.1 (213.0–1183.0)	0.047
eGFR (mL/min)	62.53 (47.53–80.77)	83.04 (47.57–86.14)	0.819
Uric acid (mg/dL)	5.0 (4.4–5.8)	6.0 (5.3–6.7)	<0.001
CRP (mg/L)	6 (5–11)	5 (5–7)	0.047
ESR (mm/h)	17 (11–27)	13 (5–22)	0.050
TSH (mlU/L)	1.50 (0.94–2.35)	1.27 (0.68–1.87)	0.292
BMI (kg/m^2^)	29.38 ± 5.13	27.31 ± 4.67	0.053
Ac (cm)	28 (26–31)	27 (26–30)	0.976
LLc (cm)	35 (33–38)	34 (31–37)	0.135
bSBP	136.54 ± 17.97	134.26 ± 19.23	0.538
bDBP	69.12 ± 9.92	70.49 ± 10.47	0.503
bMAP	91.58 ± 10.79	91.8 ± 12.44	0.923
aSBP	124.19 ± 15.99	121.09 ± 16.75	0.346
aDBP	70.22 ± 10.05	71.76 ± 10.25	0.451
aPP	51.6 (43.5–63.8)	48.7 (42.1–55.7)	0.075
aMAP	90.87 ± 10.82	89.55 ± 12.3	0.563
aHR	67.38 ± 9.57	64.61 ± 8.4	0.141
Hypertension	70 (84%)	33 (94%)	0.238
Diabetes mellitus	22 (27%)	13 (37%)	0.248
MS	33 (40%)	15 (43%)	0.754
COPD	8 (10%)	4 (11%)	0.968
HF	58 (77%)	26 (84%)	0.623
VES 13	5.29 ± 2.78	5.169 ± 3.53	0.739
ADL	5.539 ± 0.71	5.429 ± 1.06	0.957
I ADL	21.499 ± 3.56	19.529 ± 5.45	0.100
MMSE	26.619 ± 2.81	25.469 ± 5.19	0.935
CDT	8.669 ± 2.00	8.889 ± 2.29	0.244

Note 1: Continuous variables with normal distribution are presented as mean ± SD; non-normal variables are presented as median (IQR); binary variables are presented as number (percentage). Note 2: cfPWV, carotid–femoral pulse wave velocity; TC, total cholesterol; HDL-C, high-density lipoprotein cholesterol; LDL-C, low-density lipoprotein cholesterol; TG, triglycerides; FPG, fasting plasma glucose; NTproBNP, N-terminal pro b-type natriuretic peptide; eGFR, estimated glomerular filtration rate; CRP, C reactive protein; ESR, erythrocyte sedimentation rate; TSH, thyroid-stimulating hormone; BMI, body mass index; Ac, arm circumference; Llc, lower-leg circumference; bSBP, brachial systolic blood pressure; bDBP, brachial diastolic blood pressure; bMAP, brachial mean arterial pressure; aSBP, aortic systolic blood pressure; aDBP, aortic diastolic blood pressure; aPP, aortic pulse pressure, bMAP, brachial mean arterial pressure; aHR, aortic heart rate, MS, metabolic syndrome; COPD, chronic obstructive pulmonary disease; HF, heart failure; VES-13, Vulnerable Elders-13 Survey; ADL, Katz Index of Independence in Activities of Daily Living; IADL, Lawton Instrumental Activities of Daily Living Scale; MMSE, Mini-Mental State Examination; CDT, clock-drawing test.

**Table 2:** Correlation coefficients of selected parameters and cfPWV in the whole group and by gender.

Parameter	Total		Men (*n* = 35)		Women (*n* = 85)	
	r	*p*-Value	r	*p*-Value	r	*p*-Value
Age	0.341	<0.001	0.194	0.265	0.430	<0.001
HDL-C	−0.196	0.033	−0.132	0.448	−0.076	0.493
LDL-C	−0.042	0.653	0.012	0.944	−0.008	0.946
TC	−0.090	0.332	0.118	0.500	−0.095	0.393
TG	0.160	0.083	0.466	0.005	0.056	0.618
FPG	0.108	0.247	0.413	0.014	0.080	0.472
NTproBNP	0.347	<0.001	0.296	0.106	0.329	0.004
Uric acid	0.339	<0.001	0.108	0.536	0.335	0.002
CRP	0.147	0.113	0.018	0.919	0.242	0.028
ESR	0.128	0.171	0.262	0.128	0.166	0.135
TSH	0.088	0.344	0.053	0.764	0.150	0.177
eGFR	−0.212	0.021	−0.051	0.771	−0.300	0.006
BMI	0.097	0.309	0.352	0.052	0.113	0.318
Ac	0.035	0.713	0.192	0.301	0.023	0.839
LLc	−0.013	0.890	0.070	0.707	0.032	0.775
bSBP	0.443	<0.001	0.466	0.005	0.500	<0.001
bDBP	0.229	0.013	0.196	0.259	0.232	0.035
bMAP	0.374	<0.001	0.347	0.041	0.413	<0.001
aSBP	0.411	<0.001	0.450	0.007	0.471	<0.001
aDBP	0.255	0.005	0.223	0.197	0.255	0.020
aPP	0.311	<0.001	0.488	0.003	0.357	0.001
aMAP	0.353	<0.001	0.351	0.039	0.409	<0.001
AorticHR	0.009	0.922	−0.044	0.801	0.089	0.425

**Table 3:** Multivariable regression analysis coefficients.

TOTAL GROUP Variable	Unstandardized Coefficients		Standardized Coefficients		*p*-Value
	β	SE	β	SE	
Age	0.053	0.017	0.250	0.081	0.003
NTproBNP	0.000	0.000	0.119	0.085	0.165
Uric Acid	0.201	0.093	0.172	0.080	0.034
bSBP	0.037	0.007	0.398	0.077	<0.001
gender (male)	0.464	0.151	0.251	0.081	0.003
WOMEN GROUP variable	Unstandardized coefficients		Standardized coefficients		*p*-value
	β	SE	β	SE	
Age	0.059	0.021	0.276	0.098	0.006
NTproBNP	0.000	0.000	0.208	0.096	0.034
Uric Acid	0.273	0.104	0.240	0.091	0.010
bSBP	0.033	0.009	0.355	0.093	<0.001
MEN GROUP variable	Unstandardized coefficients		Standardized coefficients		*p*-value
	β	SE	β	SE	
TG	0.007	0.004	0.332	0.173	0.064
FPG	0.008	0.010	0.138	0.175	0.435
bSBP	0.031	0.011	0.394	0.143	0.010

**Table 4:** Multivariable regression—comorbidities and gender.

Parameters	Unstandardized Coefficients		Standardized Coefficients		*p*-Value
	β	SE	β	SE	
Hypertension	0.542	0.244	0.196	0.089	0.029
Diabetes Mellitus	0.508	0.162	0.279	0.089	0.002
COPD	0.048	0.238	0.017	0.086	0.842
Heart failure	0.288	0.180	0.139	0.087	0.113
Gender	0.465	0.160	0.251	0.086	0.005

**Table 5:** Analysis of multisite regional PWV by gender.

Measured Site-Dependent PWV (Regional PWV)	Women Mean [Min–Max]	Men Mean [Min–Max]	*p* Value
forehead–right toe, htPWV	9.40 [6.70–14.10]	9.34 [6.10–13.00]	0.660
forehead–left toe, htPWV	9.51 [6.10–14.10]	9.63 [6.80–14.00]	0.858
right ear–right toe, etPWV	9.41 [7.00–13.50]	9.64 [6.70–13.90]	0.951
left ear–left toe, etPWV	9.25 [6.10–13.70]	9.79 [7.00–13.30]	0.180
right finger–right toe, ftPWV	10.01 [6.10–15.30]	9.43 [6.10–14.40]	0.336
left finger–left toe, ftPWV	9.49 [6.20–13.60]	9.20 [6.50–14.40]	0.286

**Table 6:** Comparison of central and regional PWV.

TOTAL GROUP Variable	Central PWV (cfPWV) Mean [Min–Max]	Regional PWV Mean [Min–Max]	Mean Difference	*p*-Value
forehead–right toe, htPWV	9.86 [6.32–14.14]	9.38 [6.10–14.10]	0.48	0.028
forehead–left toe, htPWV		9.55 [6.10–14.10]	0.25	0.060
right ear–right toe, etPWV		9.48 [6.70–13.90]	0.44	0.015
left ear–left toe, etPWV		9.41 [6.10–13.70]	0.42	0.021
right finger–right toe, ftPWV		9.85 [6.10–15.30]	0.03	0.409
left finger–left toe, ftPWV		9.40 [6.20–14.40]	0.55	0.038
WOMEN GROUP variable	central PWV (cfPWV)	regional PWV	mean difference	*p*-value
forehead–right toe, htPWV	9.36 [6.32–13.02]	9.40 [6.70–14.10]	0.07	0.046
forehead–left toe, htPWV		9.51 [6.10–14.10]	0.12	0.166
right ear–right toe, etPWV		9.41 [7.00–13.50]	0.17	0.534
left ear–left toe, etPWV		9.25 [6.10–13.70]	0.26	0.345
right finger–right toe, ftPWV		10.01 [6.10–15.30]	0.46	0.181
left finger–left toe, ftPWV		9.49 [6.20–13.60]	0.12	0.185
MEN GROUP variable	central PWV (cfPWV)	regional PWV	mean difference	*p*-value
forehead–right toe, htPWV	10.52 [8.12–14.14]	9.34 [6.10–13.00]	1.37	0.001
forehead–left toe, htPWV		9.63 [6.80–14.00]	1.01	0.012
right ear–right toe, etPWV		9.64 [6.70–13.90]	1.07	0.005
left ear–left toe, etPWV		9.79 [7.00–13.30]	0.81	0.029
right finger–right toe, ftPWV		9.43 [6.10–14.40]	1.33	0.011
left finger–left toe, ftPWV		9.20 [6.50–14.40]	1.49	0.004

## Data Availability

Not applicable.
